# Glioblastoma Multiforme With CDKN2A, Loss of PTEN and EGFR Amplification, and Diffuse Distant Organ Metastasis Treated With Six Lines of Therapy: A Case Report and Literature Review

**DOI:** 10.7759/cureus.23796

**Published:** 2022-04-03

**Authors:** Ferit Aslan, Elif Günaydın, Fisun Yukruk, İnanç Güvenç, Onur Serdar Gençler

**Affiliations:** 1 Department of Medical Oncology, Yüksek İhtisas University Medical Park Ankara Hospital, Ankara, TUR; 2 Department of Radiology, Yüksek İhtisas University Medical Park Ankara Hospital, Ankara, TUR; 3 Department of Pathology, Dr Abdurrahman Yurtaslan Ankara Oncology Training and Research Hospital, Ankara, TUR; 4 Department of Radiology, Yüksek İhtisas University Medical Park Ankara Batıkent Hospital, Ankara, TUR; 5 Department of Neurology, Yüksek İhtisas University Medical Park Ankara Hospital, Ankara, TUR

**Keywords:** treatment resistance, extracranial, distant metastasis, systemic metastasis, glioblastoma

## Abstract

In glioblastoma multiforme (GBM) cases, the tumor usually remains limited to the central nervous system in the expected disease course. Here, we discuss the case of a 41-year-old male patient who presented with extracranial spread, which has been reported in a limited number of cases in the literature. The patient received six lines of treatment including radiotherapy with temozolomide, irinotecan-bevacizumab combination, lomustine, erlotinib, everolimus, and weekly carboplatin-paclitaxel. In addition to systemic treatment, the patient underwent radiotherapy and surgery twice. Despite presenting with features consistent with a poor prognosis and extensive multi-organ metastasis, the patient achieved an overall survival of 25 months. In our view, the clinical course of our case, the follow-up, and the treatment process will add to the literature.

## Introduction

Glioblastoma multiforme (GBM) accounts for 14.6% of adult central nervous system (CNS) primary tumors and 48.3% of primary malignant tumors [1]. The overall five-year survival in GBM is approximately 5%. World Health Organization (WHO) grade IV GBM is a highly aggressive tumor with a median survival of fewer than 15 months in adults despite optimal treatment. Age and effective and extensive surgery are the two most influential factors determining prognosis [1].

GBM is a malignant tumor whose area of ​​spread, recurrence, and progression is considered to be within the brain. The systemic spread of GBM is rare, occurring in only 0.2% to 0.4% of cases [2]. To date, fewer than 200 cases have been reported in the literature. Metastasis of GBM to the bone, lymph nodes, liver, and lungs has been reported in the literature [3]. Patients with systemic metastases to four or more sites constitute 4% of all patients with extracranial metastases. Likewise, leptomeningeal spread is rare, occurring in 2-4% of GBM patients. Most patients are young and male and have undergone prior surgery, which is believed to allow cancer cells to access the extracerebral blood and lymphatic vessels [4]. Few case reports have been previously reported in which a single tissue or organ metastasis was present.

Here, we discuss the clinical examination, treatment, and follow-up of a patient with lymph node, lung, liver, bone, and skin metastases.

## Case presentation

A 41-year-old male patient was admitted to our hospital with progressive frontal headache, nausea, and vomiting. According to the patient’s wife, the patient had displayed personality changes recently. There was no known additional disease, drug usage, or smoking history. The patient underwent magnetic resonance imaging (MRI) that showed malignancy in the left parietooccipital region, measuring 35 × 25 mm and containing an area of widespread vasogenic edema.

**Figure 1:**
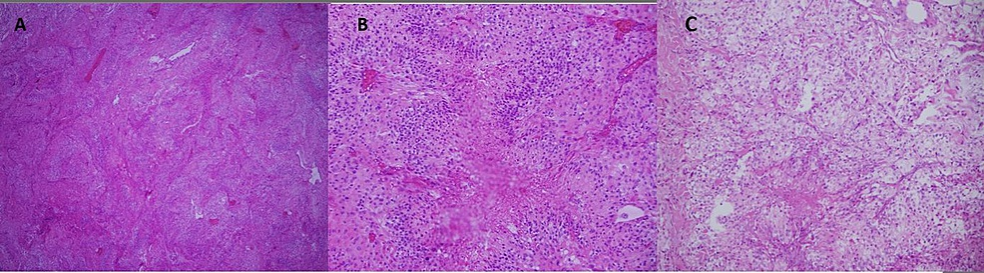
(A) High-grade infiltrative glial tumor with solid growth, branching vascular proliferation, and some thrombosed vascular structures (HE ×40). (B) Tumor cells pseudo-palisading around necrosis (HE ×400). (C) Subcutaneous tumor metastasis and necrotic pseudo-palisading tumor cells with histomorphological features similar to the primary among the dermal collagen fibers (HE ×400). HE: hematoxylin and eosin

Maximum safe tumor excision with navigation was performed along with osteoplastic craniotomy and dura mater plastic surgery. No postoperative complications were observed. On histological and immunohistochemical evaluation, O^6^-methylguanine-DNA methyltransferase (MGMT) was negative, glial fibrillary acidic protein (GFAP) was positive, isocitrate dehydrogenase-1 (IDH-1) was negative, p53 was 10%, and Ki67 was 20%, which was compatible with WHO grade IV GBM (Figure 1, Panel A, B).

**Figure 2:**
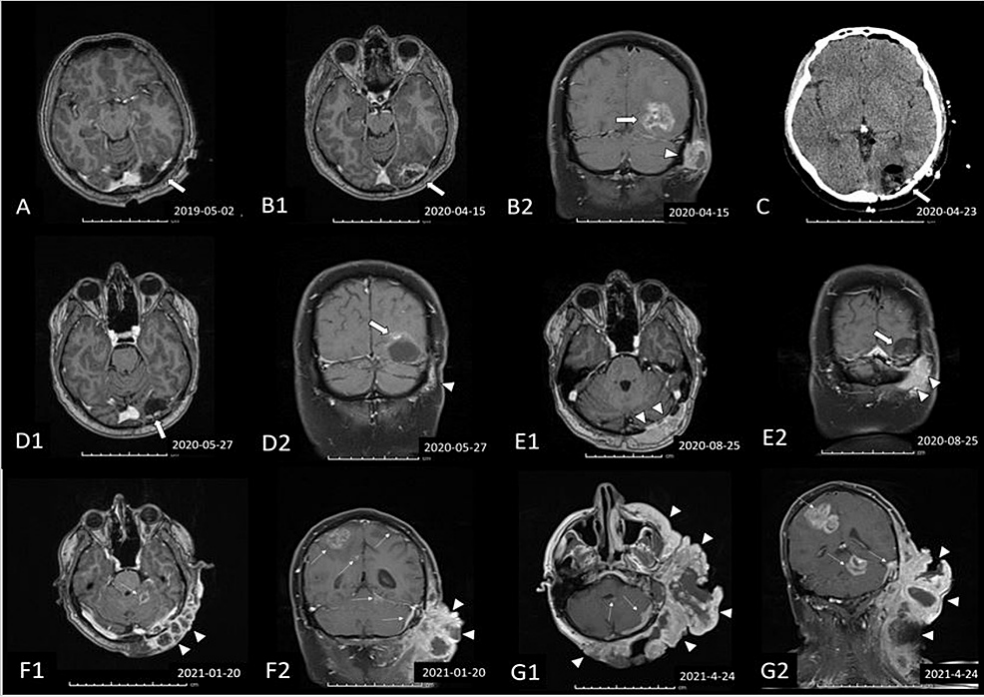
Postoperative findings after GBM (WHO grade IV) resection in the left occipital lobe (not shown). T1-weighted, gadolinium-enhanced axial and coronal MRI scans and axial CT image. (A) axial T1-weighted scan after the first surgery demonstrating a small residual tumor posterior to the surgical cavity. (B) Axial T1-weighted scan obtained approximately one year later showing residual tumor and a new scalp mass. (C) Axial CT image after the second surgery changes without residual tumor. (D) Following re-resection of the tumor, a local breakthrough through the extracranial soft tissue was observed. (E1-2 and F1-2) Axial and coronal T1-weighted scans showing significant enlargement of the extracranial mass. (G1-2) Nine months after the second surgery, new multiple contrast-enhanced masses with cystic degeneration were seen in the cerebral and cerebellar hemispheres, showing progressive disease with intra and extracranial heterogeneously enhancing masses. Arrow, GBM: arrowheads, local progression of the GBM in the extracranial soft tissue; thin arrows: intracranial metastasis GBM: glioblastoma multiforme; WHO: World Health Organization; MRI: magnetic resonance imaging; CT: computerized tomography

After the patient was given temozolomide (75 mg/m^2^/day) concurrently with adjuvant radiotherapy (total dose 60 Gy, 2 Gy in each fraction), six courses of temozolomide (200 mg/m^2^/day for five days every 28 days) were planned. However, after the sixth course of temozolomide, a thick, irregular, nodular, 18 × 30 × 28 mm contrast enhancement on the surgical resection cavity wall in the left occipital lobe was evaluated, suggestive of recurrence. Radiotherapy (a total of 25 Gy for five days) was given to the patient again. Tumor progression continued in the first month after reirradiation. The patient underwent a second maximally safe tumor resection (Figure 2).

Histological and immunohistochemical examination after the second surgery revealed GFAP-positive, IDH-1 negative, p53 30%, Ki67 40%, preserved alpha‐thalassemia mental retardation syndrome expression, and WHO grade IV GBM.

**Figure 3:**
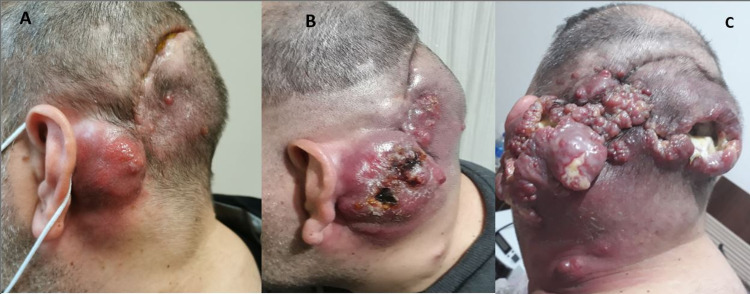
Extracranial spread of the tumor in the neck and head. (A, B) Left parieto-occipital region: a palpable 4-5 cm mass in the post-auricular region and development of left cervical LAP. (C) The final image of the destructive spread of the tumor in the neck and head before the patient’s death. LAP: lymphadenopathy

The patient’s tumor progression continued and he was administered seven cycles of bevacizumab together with irinotecan (bevacizumab 10 mg/kg, irinotecan 125 mg/m^2^ every two weeks) as the second-line treatment. After this treatment, a palpable 4-5 cm mass was observed in the left parietooccipital region and postauricular region, and left cervical lymphadenopathy (LAP) was also noted (Figures 2, 3).

**Figure 4:**
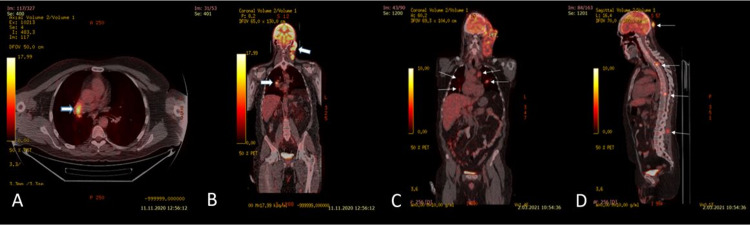
Fused FDG PET/CT scanning conducted seven months after the reoperation revealing an area of hypermetabolism in the right hilar lymph node (arrow) (A) and showing the local progression of the GBM in the left collar region (arrows) (B). (C, D) Repeat fused FDG PET/CT scanning in identifying disseminated metastatic spread to the lungs, mediastinal lymph nodes, scalp, vertebrae, and surrounding paravertebral soft tissues (thin arrows). FDG: Fluorodeoxyglucose; PET-CT: positron emission tomography-computed tomography

The patient’s positron emission tomography-computed tomography imaging revealed that the tumor had spread from the cervical trace to the mediastinal lymph nodes (Figure 4).

Tru-cut biopsy from left cervical LAP was interpreted in favor of GBM metastasis when evaluated with previous pathologies (Figure 1, Panel C). The patient with tumor progression was given two courses of lomustine (110 mg/m^2^ every six weeks) in the third step. After two cycles, the patient presented with an increasing headache, a mass with serous discharge reaching 6 cm behind the ear, multiple cervical LAPs, and subcutaneous swellings on the anterior chest and back. Lung, liver, mediastinal, cervical conglomerate LAP, and subcutaneous metastases were detected in PET-CT taken for systemic disease evaluation (Figure 4).

There were no appropriate clinical studies available for the young patient who had an ECOG performance score of 1 and treatment expectation. One of the multiple gene analyses was performed on the patient.

Next-generation sequencing) based on Foundation One ®CDx was performed from the patient’s tumor brain tissue and metastatic tissue. No microsatellite instability was detected. Tumor mutation load was found to be 3. Genetic changes of epidermal growth factor receptor (EGFR) (A289V, amplification, S7681-subclonal, H773R-subclonal, V774M) and phosphatase and tensin homolog (PTEN) (Splice site 209+1-209+2delGT), cyclin-dependent kinase inhibitor 2A/B (CDKN2A/B) loss, mutY DNA glycosylase (MUTYH) 281L, and telomerase reverse transcriptase (TERT) promoter -124>T were detected.

The patient was started on weekly carboplatin and paclitaxel (80 mg/m^2^ paclitaxel, two AUC carboplatin) in the fourth step. The patient’s disease that achieved a clinical partial response in the first month of the treatment progressed radiologically and clinically in the second month (during the eighth week of the treatment). Subsequently, although erlotinib and everolimus were given based on genetic test results, there was no response. In the following period, massive bleeding occurred from the mass lesion seen outside the head, and the patient died due to intracranial diffuse edema and bleeding during the follow-up in the intensive care unit. Contrary to expectations and literature, the patient with a poor prognosis achieved a total survival time of 25 months from diagnosis.

## Discussion

GBM is an aggressive tumor that usually spreads to adjacent brain tissues via white matter. Despite its local and adjacent aggressiveness, the extracranial spread is rare. In their systematic review, Pietschmann et al. reviewed 109 eligible studies published from 1928 to 2013 and found a total of 150 GBM patients diagnosed with systemic metastases [5,6]. Since then, 17 more cases have been reported. In these case reports, the mean time to metastasis was eight months, and the mean survival time after metastasis was six months [7,8]. The survival time after detection of metastases in four or more regions has been reported as one month in the literature [5].

In a review of 128 patients with extracranial metastases, Piccirilli et al. reported that the most common metastases were lungs (n = 44, 34.4%) and bones (n = 29, 22.9%). Moreover, vertebral involvement was the most common among bones [9].

Although the rarity of metastases from GBM is unclear, some causes have been suggested. First, the short life expectancy of the patient with GBM eliminates the possibility of detectable GBM metastases. Second, the blood-brain barrier (BBB) ​​plays a physically important role against the migration of brain tumor cells into the bloodstream. Another reason is that the lack of extracellular matrix component and lymphatic system in the brain makes it difficult for the tumor cell to metastasize to outer spaces [10]. It may result from subcutaneous implantation of tumor cells during the surgical removal of the tumor or cell escape through the dura toward the skin along the excision/biopsy pathway [10-12].

The risk of extraneural metastases is unclear. However, many neuro-oncologists accept that young age, long life span, frequent recurrences, high-grade histology, and sarcomatous components may increase this risk. It is widely accepted that neurosurgical operations associated with the opening of brain vessels may damage the BBB which causes hematogenous extraneural spread of the brain tumor [5,12]. It is also accepted that dura mater provides an important barrier to extracranial spread. Skin metastases after craniotomy have been reported in approximately 20 patients in the literature [12].

MYCN or MYC gene amplifications were found in 30-40% of cases with cerebrospinal fluid spread [13]. Overall, 90% of GBM cases are IDH-1/IDH-2 wild-type. IDH wild-type GBM often shows molecular changes such as EGFR amplification and chromosome 10 losses. It has been stated that extracranial spread may be more in patients with IDH-1/IDH-2 wild-type, 1p/19q deletion, and unmethylated MGMT promoter [12,13].

The Cancer Genome Atlas study showed that in the whole-exome sequence of GBM tumors, PTEN, TP53, EGFR, phosphatidylinositol-4,5-bisphosphate 3-kinase catalytic subunit alpha (PIK3CA), phosphoinositide-3-kinase regulatory subunit 1 (PIK3R1), neurofibromatosis 1 (NF1), retinoblastoma protein 1 (RB1), IDH-1, and platelet-derived growth factor receptor alpha (PDGFRA) genes were significantly mutated. EGFR alterations can be seen in 54% of GBM cases. EGFR amplifications are seen in 48% of cases with GBM, and mutations are seen in 21% [14]. There is no clear relationship between EGFR amplifications and survival in GBM. In our patient, we could not get a response from the erlotinib treatment given due to EGFR amplification.

## Conclusions

The reasons for the extracranial spread of a highly aggressive tumor such as GBM are not yet clear. Although all patients operated on for GBM have impaired anatomical integrity, including dura mater and BBB due to extracranial operation, very few patients have extracranial metastases. Although extracranial spread is likely multifactorial, we think that molecular and genetic changes in the tumor play an important role. The genetic and molecular classification of GBM in future studies, such as other solid organ tumors, will provide clinicians with more comprehensive information about the behavior of the tumor and treatment planning. Our patient achieved an overall survival of just over two years despite poor prognostic features and poor response to treatment. The most important reason for this is the extracranial spread pattern of the tumor, not intracranial. Again, the cause of mortality in this patient appears to be due to intracranial spread in the later stages of the disease. A moderate response to weekly carboplatin and paclitaxel is an important outcome when there is systemic spread. There was no response to the drugs given for this target. Consequently, in our view, this case will make an important contribution to the GBM literature in terms of its course, treatment, spread, and molecular genetic features.
